# Actin and Microtubules Differently Contribute to Vacuolar Targeting Specificity during the Export from the ER

**DOI:** 10.3390/membranes11040299

**Published:** 2021-04-20

**Authors:** Monica De Caroli, Fabrizio Barozzi, Luciana Renna, Gabriella Piro, Gian-Pietro Di Sansebastiano

**Affiliations:** 1 DISTEBA (Department of Biological and Environmental Sciences and Technologies), University of Salento, Campus ECOTEKNE, 73100 Lecce, Italy; monica.decaroli@unisalento.it (M.D.C.); barozzi.fabrizio@virgilio.it (F.B.); gabriella.piro@unisalento.it (G.P.); 2 Department of Plant Physiology, Faculty of Biology, Chemistry and Earth Sciences, University of Bayreuth, Universitätsstraße 30, D-95447 Bayreuth, Germany; 3 Department of Biology, University of Florence, 50121 Firenze, Italy; luciana.renna@unifi.it

**Keywords:** cytoskeleton, vacuole, endoplasmic reticulum, traffic, tubulin, actin, cytochalasin D, Taxol

## Abstract

Plants rely on both actin and microtubule cytoskeletons to fine-tune sorting and spatial targeting of membranes during cell growth and stress adaptation. Considerable advances have been made in recent years in the comprehension of the relationship between the trans-Golgi network/early endosome (TGN/EE) and cytoskeletons, but studies have mainly focused on the transport to and from the plasma membrane. We address here the relationship of the cytoskeleton with different endoplasmic reticulum (ER) export mechanisms toward vacuoles. These emergent features of the plant endomembrane traffic are explored with an in vivo approach, providing clues on the traffic regulation at different levels beyond known proteins’ functions and interactions. We show how traffic of vacuolar markers, characterized by different vacuolar sorting determinants, diverges at the export from the ER, clearly involving different components of the cytoskeleton.

## 1. Introduction

Eukaryotic cells are characterized by a complex and dynamic endomembrane system, which further evolved with multicellularity to assure specialized functions. Core components of the eukaryotic endomembrane system are broadly conserved, but there have been substantial diversifications to satisfy all requirements of endomembrane trafficking. In particular, plant cells diversify from animal cells at the level of export from the endoplasmic reticulum (ER) to the complex organization of the Golgi apparatus distinguishable in dictyosomes. Furthermore, the presence of a large central vacuole forces the distribution of all membranous compartments to the periphery of the cell, increasing traffic distance and complexity.

The cytoskeleton represents a dynamic scaffold maintaining the subcellular localization patterns of membranous compartments in specialized cell types, and controls growth, development and physiological rapid responses [1,2]. It has been established that differently from metazoans, long-distance transport of endomembrane components in plants mainly relies on actin bundles and is mediated by myosin XI motor proteins [3]. Myosin XI motor proteins’ maximum speeds exceed those of microtubule-associated kinesin motors [4], and they are more effective at providing the driving force of cytoplasmic streaming, which assures the distribution of endomembrane compartments and cytosolic components in cells that are often much bigger than in other organisms. Endomembrane compartments are in close communication with each other through dynamic interactions mediated by multiprotein complexes. In vesicular traffic, membrane-bound Rab GTPases can recruit other proteins involved in compartment motility such as motor proteins, tethering factors and SNAREs (N-ethylmaleimide-sensitive factor adaptor protein receptors) [2], but the microtubule-based transport, for a long time unnoticed in plant cells, is an essential player in traffic too [5,6]. The necessary spatiotemporal control of traffic must depend on the trans-Golgi network/early endosome (TGN/EE), a morphologically complex post-Golgi compartment considered to be the hub for both secretory and endocytic pathways [7]. The TGN/EE arises through maturation of the terminal trans-Golgi cisternae and acquires different functional subdomains characterized by partially overlapping sets of proteins [8]. A TGN undergoing this maturation may also be named a Golgi-associated TGN (GA-TGN), when still part of the dictyosome, or a Golgi-independent TGN (GI-TGN), when it is out of the Golgi matrix [7,9]. Some secretory pathways are able to bypass the TGN/EE [10], as well as some others that bypass the Golgi from which the TGN/EE is originated [11], but most secreted cargo molecules, including membrane-bound and secreted proteins and cell wall matrix polysaccharides, pass through the TGN/EE. The most studied cargo molecules have been characterized as being sorted through the Golgi apparatus; thus, when sorting bypasses this organelle, the process is considered “unconventional” [12]. Recent research works on the TGN/EE’s role in protein traffic and its relationship with the cytoskeleton have mainly focused on the transport to and from the plasma membrane [6,13] or during cell division [14].

Here, we investigate the role of actin and microtubules in the traffic of two vacuolar markers sorted by different secretory pathways [15,16]. We induced the alteration of the different cytoskeleton components and compared the effects on vacuolar and Golgi markers. The vacuolar markers consist of a fluorescent protein carrying either the sequence-specific vacuolar sorting determinant of Aleurain at the N-terminus (e.g., Aleu-GFP) or the C-terminal vacuolar sorting determinant of tobacco chitinase A (e.g., RFP-Chi). The characterization of the pathway responsible for the sorting of proteins with chitinase A signal (Chi) is still poor, even if it seems largely independent from the Golgi [16]. This marker in its GFP-based version has been related to the SNARE Vti12 [17], to the putative receptor RMR1 (Receptor Membrane RING-H2) [11] and to the ER membrane export labeled by the aquaporin NIP1.1 [18]. We show that, while Aleu-GFP is sorted by the Golgi, the RFP-Chi marker is exported from the ER labeling discrete independent punctate structures. Thanks to the complementary observation of the Golgi markers ERD2-YFP, CslA2-GFP and ST52-mCherry, new considerations can be drawn about ER export and TGN maturation.

## 2. Materials and Methods

### 2.1. Preparation of Genetic Constructs

The constructs expressing the fluorescent markers Aleu-GFP [15], ERD2-YFP [19], CslA2-GFP [20] and ST52-mCherry [21] were previously described. RFP-Chi is described here for the first time. The open reading frames of RFP were amplified from the existing template [22] with specific primers to include the attB1 Gateway attachment sites (forward primer, GGG GAC AAG TTT GTA CAA AAA AGC AGG CTT TAT GAA GAC TAA TCT TTT TC) and the Chi signal (reverse primer, TTA CAT AGT ATC GAC TAA AAG ATC GGC GCC GGT GGA GTG GCG GCC CTC GG). The PCR product was gel-purified and used as a template for a second PCR using the forward primer used before and another reverse primer (GGG GAC CAC TTT GTA CAA GAA AGC TGG GTA TTA CAT AGT ATC GAC TAA AA) to include the attB2 Gateway attachment sites. A subsequent BP clonase reaction in pDONR221 (Invitrogen) yielded an Entry clone, which was verified via sequencing. A subsequent LR clonase reaction (Invitrogen) using the pDEST plasmid pK2GW7 permitted the obtainment of the final construct RFP-Chi.

### 2.2. Plant Material: Protoplast Preparation and Transformation

*Nicotiana tabacum* cv SR1 plants were grown as reported in [23]. Tobacco leaf protoplasts were prepared and transformed as described in [24]. Equal quantities (20 μg) of each plasmid were used for the co-localization and co-expression experiments. The fluorescent pattern of thousands of cells was observed before imaging.

### 2.3. Drug Treatments

Six hours after transformation, protoplasts transiently expressing fluorescent constructs were incubated with or without 80 µM Cytochalasin D (Cyt D; Merck KGaA, St. Louis, MO, USA) or with and without 10 µM Taxol (Tax; Tocris, Bristol, UK) for the time reported in each figure. Drugs were dissolved in dimethyl sulfoxide (DMSO) in a × 1000 stock solution.

### 2.4. Confocal Laser Scanning Microscopy

Protoplasts transiently expressing fluorescent constructs were observed by a laser scanning confocal microscope (LSM 710 Zeiss, Munich, Germany) in their culture medium at different times after transformation. To detect GFP fluorescence, a 488 nm argon ion laser line was used, and the emission was recorded with 505–530 nm filter set; RFP was detected with a 560–615 nm filter set after He-Ne laser excitation at 543 nm, while chlorophyll epifluorescence was detected with the filter >650 nm. The power of each laser line, the gain and the offset were identical for each experiment so that the images were comparable. Appropriate controls were performed to exclude the possibility of crosstalk between the two fluorochromes before image acquisition. Chlorophyll epifluorescence is shown in some figures to rule out the possibility of fluorescence derived from chlorophyll bleaching.

### 2.5. Data Analysis

The quantitative evaluation of drugs’ effects on the fluorescent pattern of RFP-Chi was carried out by counting the magenta dots not colocalizing with the GFP marker. Confocal images (1.4 µm thickness) of 20 different protoplasts, collected in 3 independent experiments (different preparations and different starting plant material), were visually analyzed (*n* > 6). Results are presented as the mean value with their standard deviation (SD) as reported in each figure. P-values were calculated using one-way ANOVA with Tukey’s post-test. Graphical and statistical analysis was performed using Graph-Pad PRISM (Graph-Pad software, San Diego, CA, USA).

### 2.6. Network Analysis of In Silico Interactions

In silico analysis was performed using the software Cytoscape 3.8.1 [25] retrieving data from the BAR database [26]. TAIR id of VSR1 (At3g52850), RMR1 (At5g66160), VTI11 (At5g39510) and VTI12 (At1g26670) were used as initial input. Only elements of the cytoskeleton, SNAREs and aquaporins were selected to obtain the final network.

## 3. Results

### 3.1. Golgi-Independent ER Export Leads to an Uncharacterized Intermediate Compartment

**Figure 1:**
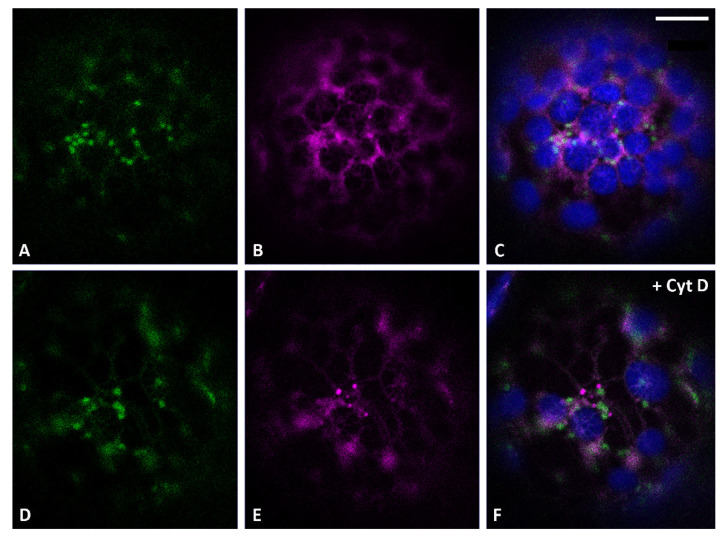
Confocal images of tobacco protoplasts transiently expressing ERD2-YFP (in green) and tobacco chitinase A (RFP-Chi) (in magenta). (**A**) ERD2-YFP distribution, (**B**) RFP-Chi distribution and (**C**) merge of the two emissions plus the epifluorescence of chlorophyll (in blue) in control conditions. (**D**) ERD2-YFP distribution, (**E**) RFP-Chi distribution and (**F**) merge of the two emissions plus the epifluorescence of chlorophyll (in blue) 3 h after treatment with actin depolymerizing agent cytochalasin D (Cyt D) 80 µM. Scale bar = 10 µm.

It was previously shown that the construct GFP-Chi was exported from the ER to the vacuole by labeling discrete independent punctate structures, not yet characterized, that only partially co-localized with the Golgi [15,16,27]. In this study, *Nicotiana tabacum* protoplasts were transiently co-transformed with the *cis*-Golgi marker ERD2-YFP [19] and RFP-Chi, an RFP-based marker with a fluorophore more stable than GFP at low pH [28,29]. We observed that the intermediate compartments labeled by RFP-Chi were more visible (Figure 1A–C) with respect to compartments highlighted by the GFP tagged form of the marker [15,16,27]. In order to facilitate imaging of small compartments disturbed by the Golgi’s high motility, the same co-transformed protoplasts were treated with the actin depolymerizing agent cytochalasin D (Cyt D), which abolishes Golgi movement [30]. A high concentration of 80 µM was used to guarantee an immediate effect on tagged compartments, since the use of 40 µM [31] and 60 µM required about 1 h to fully abolish Golgi movements. RFP-Chi-labeled compartments independent from ERD2-YFP-labeled Golgi were clearly observed from 2 to 12 h after treatment (Figure 1D–F).

### 3.2. Actin Filaments and Microtubules Differently Affect Golgi-Dependent and Golgi-Independent Traffic

Having successfully and consistently highlighted the independent compartments with the use of RFP-Chi, we then aimed to spatially locate within the secretory pathway these structures relating them with the *trans* cisternae of the Golgi and the maturating TGN into a GI-TGN.

**Figure 2:**
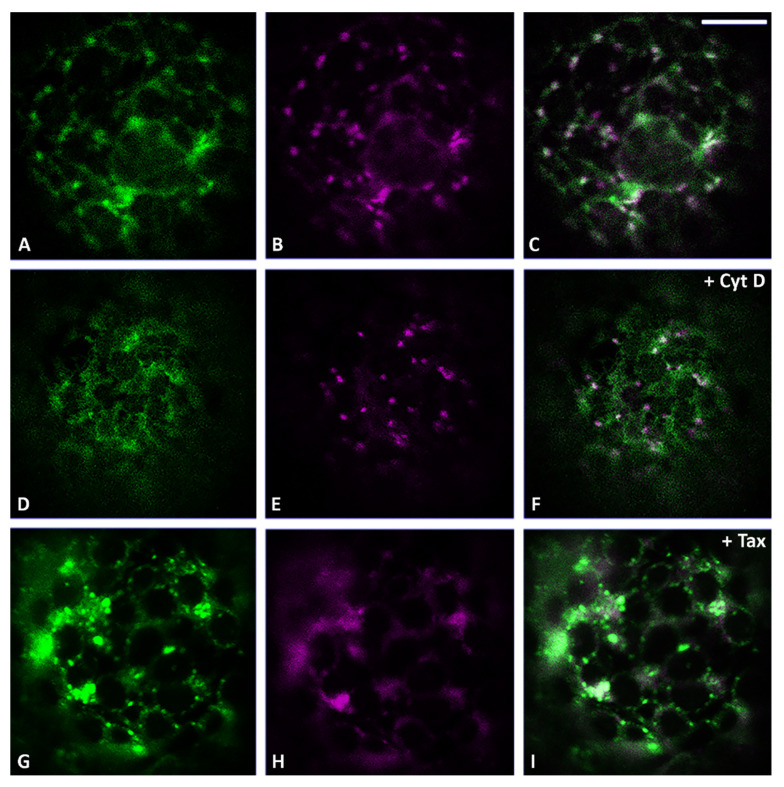
Confocal images of tobacco protoplasts transiently expressing CslA2-GFP (in green) and ST52-mCherry (in magenta). (**A**) CslA2-GFP, (**B**) ST52-mCherry and (**C**) merge distributions in control conditions. (**D**) CslA2-GFP, (**E**) ST52-mCherry and (**F**) merge distributions in the presence of Cyt D. (**G**) CslA2-GFP, (**H**) ST52-mCherry and (**I**) merge distributions in the presence of Taxol (Tax). Scale bar = 10 µm.

We initially tried to understand and clarify the exact distribution pattern of two markers: CslA2-GFP, a Golgi localizing protein [20] distributed in all the cisternae from the *cis* to the *trans* side (Figure 2A), and ST52-mCherry [21,32], better known to preferentially label *trans*-Golgi cisternae (Figure 2B). The co-localization analysis showed only a partial overlapping between the organelle regions labeled by CslA2-GFP and the ones labeled by ST52-mCherry (Figure 2C), confirming the main localization of CslA2-GFP to the Golgi and suggesting that ST52-mCherry, even if present in the *trans* side of the Golgi, was probably also cycling through the TGN and could be seen separated from Golgi markers.

When we blocked the actin-dependent movement using Cyt D in these samples, we observed a different impact on the localization of CslA2-GFP and ST52-mCherry. Indeed, while CslA2-GFP was retained in the ER (Figure 2D,F), ST52-mCherry remained distributed in small compartments (Figure 2E,F), similarly to control conditions (Figure 2B).

On the contrary, blocking the microtubule-dependent movement using Tax (10 μM) [33], a tubulin depolymerization agent, we observed an inverted situation: CslA2-GFP distribution was affected in a limited way (Figure 2G), but ST52-mCherry distribution was heavily compromised, causing marker relocation in aberrant aggregates (Figure 2H). This treatment clearly had an impact on the TGN organization more than on CslA2-GFP distribution to the Golgi.

**Figure 3:**
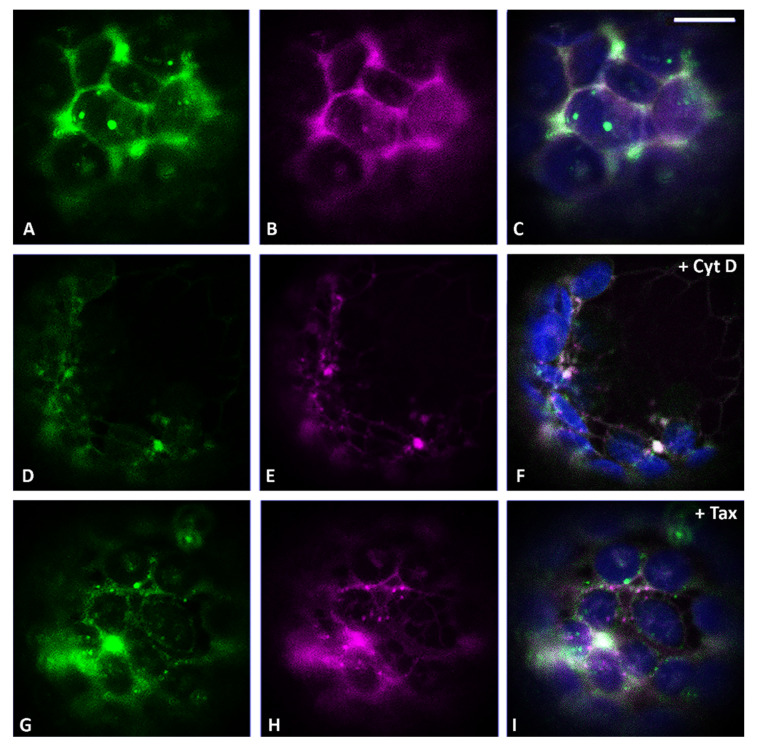
Confocal images of tobacco protoplasts transiently expressing CslA2-GFP (in green) and RFP-Chi (in magenta). (**A**) CslA2-GFP distribution, (**B**) RFP-Chi distribution and (**C**) merge of the two emissions plus the epifluorescence of chlorophyll (in blue) in control conditions. (**D**) CslA2-GFP, (**E**) RFP-Chi and (**F**) merge distributions in the presence of Cyt D. (**G**) CslA2-GFP, (**H**) RFP-Chi and (**I**) merge distributions in the presence of Tax. Scale bar = 10 µm.

Having observed a selective impact of the two cytoskeleton inhibitors on the behavior of the two different Golgi markers, we decided to use this approach to compare the drugs’ effect on RFP-Chi and CslA2-GFP distribution patterns to understand the formation and motility of the characteristic punctate structures highlighted by RFP-Chi. In control conditions, CslA2-GFP was distributed to the ER and Golgi apparatus (Figure 3A), while RFP-Chi labeled mostly the ER (Figure 3B). We observed co-localization in the ER but a poor signal of RFP-Chi in Golgi labeled by CslA2-GFP (Figure 3C).

After treatment with Cyt D, both markers’ distribution was affected. CslA2-GFP was partially retained in the ER (Figure 3D), while RFP-Chi left the ER more efficiently (Figure 3E). Co-localization of RFP-Chi and CslA2-GFP was limited to larger compartments while the smaller (presumably Golgi) were labeled by CslA2-GFP only (Figure 3F).

After treatment with Tax, both the markers partially co-localized in the larger intermediate compartments but not in the small dots (Figure 3G–I). Prolonged treatments caused similar effects both with Cyt D (Figure S1) and Tax (Figure S2). Larger intermediate compartments were formed and persisted as separated entities.

Since RFP-Chi-labeled compartments proved independent from Golgi but merging with Golgi markers, we hypothesized they are ER Microtubule-related Export Compartments (ERMEC).

### 3.3. RFP-Chi Transits through Intermediate Compartments Different from Those Highlighted by Aleu-GFP

**Figure 4:**
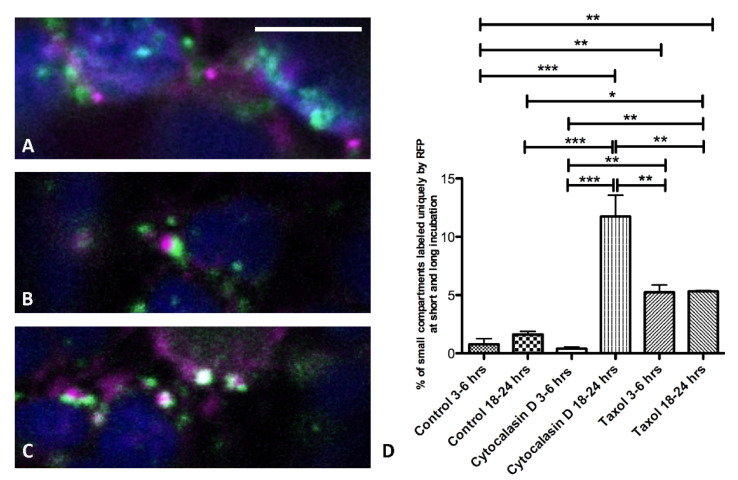
Confocal images of small compartments labeled by Aleurain at the N-terminus (Aleu-GFP) (green) and RFP-Chi (magenta) in co-transformed tobacco protoplasts. Examples of differently labeled (**A**) independent, (**B**) associated and (**C**) overlapping compartments; (**D**) counting of magenta compartments (only labeled by RFP-Chi) in two time intervals, early (3–6 h) and late (18–21 h) time intervals (*n* > 6). Error bars indicate S.E.M; p-values are represented by asterisks when significantly different from the corresponding control: * 0.01 < *p* < 0.05; ** 0.001 < *p* < 0.01; *** *p* < 0.001.

The RFP-Chi marker based on the chitinase A targeting signal has often been used in comparative studies of the trafficking pathway to the vacuole [27], as opposed to Aleu-XFP, which is based on the Aleurain sequence-specific vacuolar sorting determinant [15,34]. To deeply investigate and compare the pathways followed by these two different markers, we again used the cytoskeleton inhibitors Cyt D and Tax. We analyzed, in particular, the first sorting steps of Aleu-GFP and RFP-Chi. Following this approach, it was possible to observe that Aleu-GFP and RFP-Chi were exported from the ER through intermediate compartments appearing as small punctate structures in control conditions and upon treatments, and which can be seen as separated entities (Figure 4A), partially associated (Figure 4B) or overlapping (Figure 4C). These compartments evolved in more complex patterns with larger pro- or pre-vacuoles (Figure S3D–F also shows the effect of inhibitors on long treatment periods). We hypothesized that the smaller compartments were formed early in the sorting pathway since they were more abundant in the first 12 h after transient transformation.

Cyt D and Tax treatments induced a diversified effect on the merging of these small compartments. To describe such an effect, the most relevant parameter was the variation of the intermediate compartments exclusively labeled by RFP-Chi. These compartments were generally rare (around 2% of total compartments), but, in some circumstances, they were seen to increase significantly. The observations were performed at two distinct time intervals to distinguish an early effect on compartments formation (3–6 h) and a late effect on the merging of the compartments, 18 h after treatment (Figure 4D and Figure S3G–O).

### 3.4. Definition of the Protein Interaction Network Leading to Alternative Vacuolar Sorting Pathways

In order to clarify the different intermediate compartments labeled by the organelle markers used in this work in the presence of Cyt D and Tax, an in silico protein interaction network study was additionally performed, searching the connections between markers and cytoskeleton components. The different targeting peptides characterizing the markers were previously associated with different receptors. The Aleurain signal carried by Aleu-GFP was associated with VSR1 [35], while the chitinase A signal carried by RFP-Chi was hypothesized to be associated with RMR1 [11]. Similar markers were also directly related to specific SNAREs belonging to the same gene family, Vti11 and Vti12 [17]. An interaction network was then generated starting from these four proteins to verify the interaction with cytoskeleton components. A total of 91 interactors and 125 interactions were found.

**Figure 5:**
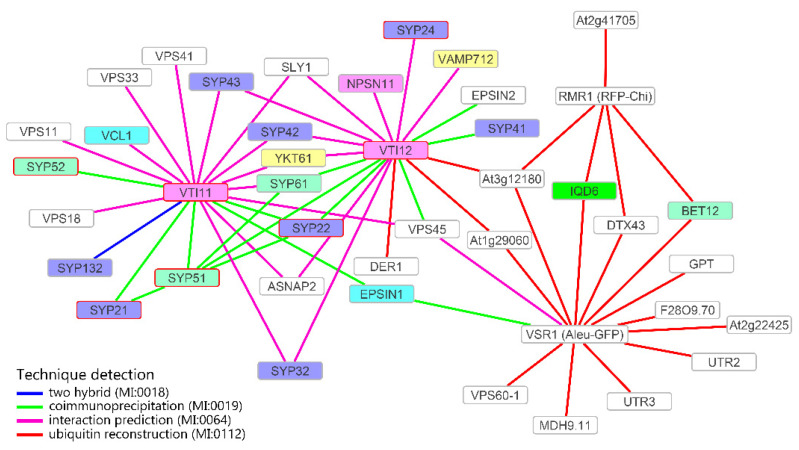
Interaction network of Vti11, Vti12, RMR1 and VSR1. Only N-ethylmaleimide-sensitive factor adaptor protein receptors (SNAREs) and proteins related to the cytoskeleton are shown. Interactors highlighted in violet are QaSNAREs, in pink QbSNAREs, in turquoise QcSNAREs, and in yellow R-SNAREs. Interactors with a red border are SNAREs involved in the formation of a vacuolar complex. Actin interacting proteins are highlighted in cyan, and tubulin interacting proteins are highlighted in green. Data retrieved from the BAR database (http://bar.utoronto.ca, accessed on 21 October 2020).

Among interactors, we selected elements of the cytoskeleton and SNAREs evidently involved in traffic, and aquaporins that may have uncharacterized structural functions. The selection was inspired by our recent observation that the interaction between the SNARE AtSYP51 and the aquaporin AtNIP1.1 may regulate the crosstalk between Golgi-mediated and ER direct transport to the vacuole [18]. The network generated by the selected proteins (42 nodes and 59 interactions) strongly supports the experimental data shown so far (Figure 5).

Vti11, found to be more important for Aleu-GFP sorting [17], is the only protein putatively interacting with an actin-related protein, the actin-binding protein VCL1 (AT2G38020) [36]. The assumption that Aleu-GFP sorting occurs through the Golgi apparatus thanks to the interaction with VSR1 is widely accepted. VSR1 interacts with Vti11 through the common interactors EPSIN1 [37] and VPS45 [17,38], while contacts with RMR1 are indirect.

Vti12, found to be more important for GFP-Chi sorting [17], interacts with RMR1 through the protein with unknown function At3g12180 [26] and then through RMR1 with the tubulin-related gene IQD6.

### 3.5. Both Actin Filaments and Microtubules Are Required for the Correct Organization of TGN

**Figure 6:**
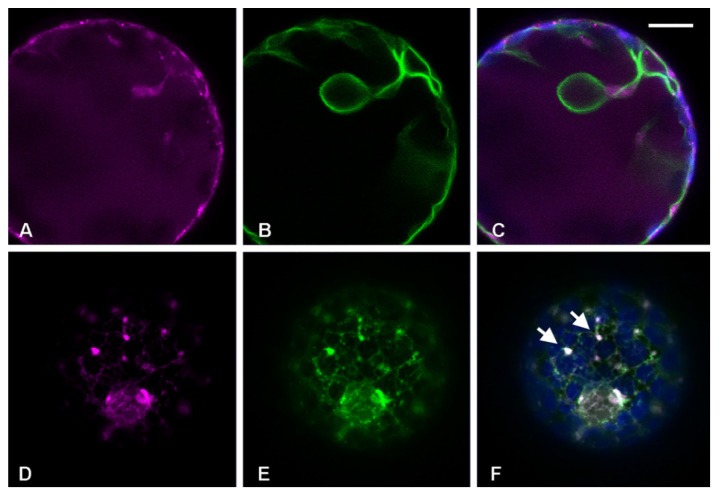
Tobacco protoplasts transiently co-expressing RFP-Chi (in magenta) and GFP:SYP51 (in green). (**A**) After more than 24 h, RFP-Chi labeled the central vacuole and (**B**) GFP:SYP51 labeled the tonoplast, (**C**) with no evident co-localization. Observing the cells during the first few hours of expression (five hours in the selected image), both (**D**) RFP-Chi and (**E**) GFP:SYP51 labeled small intermediate compartments, and (**F**) the two markers co-localized in the larger (see arrows) ones. Scale bar = 10 µm.

Since Aleu-GFP and RFP-Chi intermediate compartments can merge before reaching the final destination, a *post*-Golgi small compartment must be involved. Since we have seen that the distribution of the *trans*-Golgi marker ST52-mCherry can be selectively affected by Tax (Figure 2H), we hypothesized microtubules may influence maturation of Golgi-independent TGNs and investigated this aspect in more detail. GFP-SYP51 was seen transiting through a TGN to reach the tonoplast (Figure 6A–C) [17], but during the first few hours of expression, it labeled larger intermediate compartments co-labeled by RFP-Chi (Figure 6D–F arrows).

**Figure 7:**
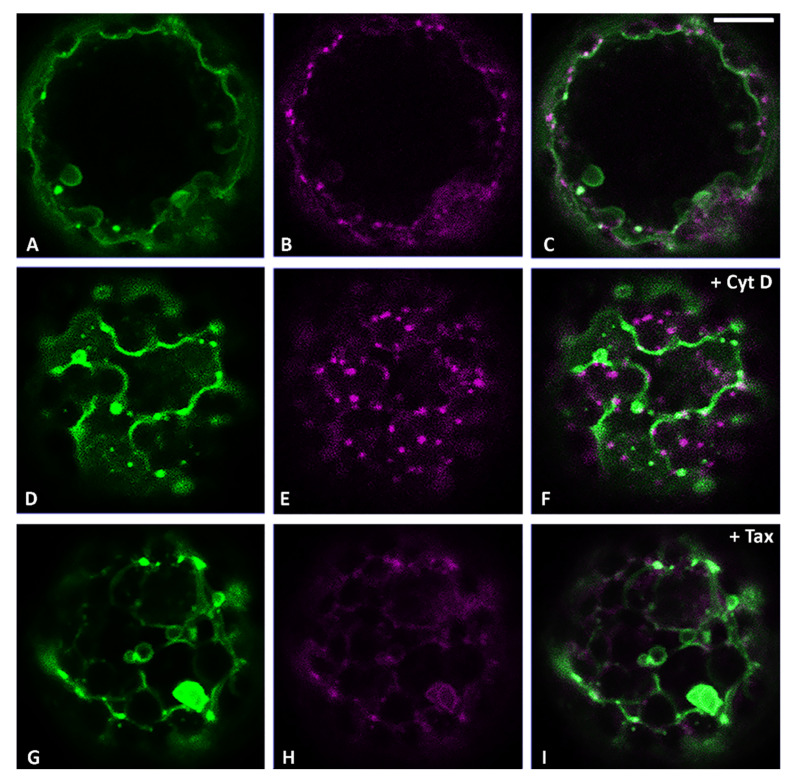
Confocal images of tobacco protoplasts transiently expressing GFP:SYP51 (in green) and ST52-mCherry (in magenta). (**A**) GFP:SYP51, (**B**) ST52-mCherry and (**C**) merge distributions in control conditions. (**D**) GFP:SYP51, (**E**) ST52-mCherry and (**F**) merge distributions in the presence of Cyt D. (**G**) GFP:SYP51, (**H**) ST52-mCherry and (**I**) merge distributions in the presence of Tax. 8 h drug treatment. Scale bar = 10 µm.

To check if SYP51 may cross-path with RFP-Chi in the maturing GI-TGN, we co-expressed GFP:SYP51 and the *trans*-cisternae Golgi marker ST52-mCherry in protoplasts applying the cytoskeleton inhibitors, and found that GFP:SYP51 distribution was affected by Cyt D (Figure 7D) but much more by Tax (Figure 7G), which induced redistribution on larger membranous multivesicular structures also for ST52-mCherry (Figure 7E,H and Figure 2).

## 4. Discussion

ER export toward vacuoles is a complex topic because many mechanisms have been described in very different experimental systems, some related to vacuole biogenesis, others to vacuolar targeting [39]. Here, we investigate the relationship between this traffic step and the cytoskeleton, evidencing the involvement of the TGN/EE.

We used the well-established in vivo approach of protoplast transient transformation to investigate how traffic of vacuolar markers, characterized by different vacuolar sorting determinants, diverges at the very beginning of its path, the export from the ER. We collected data supporting the idea that the difference also resides in the involvement of cytoskeleton components.

Vacuolar transport in plant cells is regulated by factors and mechanisms that are still largely unknown. The trafficking of vacuolar sorting receptors and the trafficking of a large amount of membrane proteins addressed to the vacuole appear to follow independent mechanisms [12]. Along the ER-to-vacuole route, intermediate compartments, whose nature is still elusive because of partially overlapping with the Golgi-mediated traffic, have been identified [7,16]. The use of inhibitory drugs, Cyt D and Tax, affecting the cytoskeleton organization helps to distinguish small compartments labeled by different vacuolar markers, thus promoting further studies on the role of cytoskeleton components. Their role is evident but it has never been investigated at the initial step of export from the ER.

The actin cytoskeleton surrounds the vacuole and contributes to the regulation of vacuolar size [40], possibly integrating extracellular sensing and intracellular control of vacuolar volume. Auxin-induced constrictions of the vacuole also depend on the actin cytoskeleton. In actin and myosin mutants, auxin-induced changes in vacuolar morphology, cell-size restriction and inhibition of root growth were all largely abolished [40]. In mammalian cells, members of the homotypic fusion and protein sorting (HOPS) complex interact with the actin cytoskeleton [41]. The plant homolog counterpart mediates homotypic vacuole fusion [42], and if the actin interaction was confirmed, would partly explain the actin dependency of auxin-induced vacuolar changes.

Due to the close proximity between tonoplast and actin filaments, it has been suggested that there might be a direct physical connection [43]. Indeed, GFP fusion protein labeling of actin filaments showed that proteins of the plant-specific Networked (NET) family possess an actin-binding domain and are membrane-associated [44]. One member in particular, NET4A, binds actin and overlaps with the tonoplast.

Microtubules and kinesins have also recently been found to play a role in vacuolar trafficking [45]. Contributions of both actin and microtubule cytoskeletons have also been described in the context of organizing the edge-directed secretory route mediated by RabA5c. Microtubules might also be involved in controlling trafficking rates to different subcellular domains as revealed by PIN2 recycling [46].

The large variety of “*post*-Golgi” compartments must also be under the control of the cytoskeleton. Endosomes characterized by sorting nexin 1 (SNX1), a well-known retromer complex component, are stabilized through the interaction with microtubule-associated protein CLIP-associated protein (CLASP) [47,48]. Another traffic mechanism involving both microtubules and actin filaments is the formation of the phragmoplast [14,49,50]. Additionally, microtubules were found to be linked to TGN/EE through TGNap1, which operates through the interaction with YIP4A/B and RAB-H1b regulating vesicle trafficking, TGN biogenesis and function [6].

Another example of the diversified involvement of actin filaments and microtubules in traffic is the cellulose synthase complex (CSC) recycling from the PM to MASCS [7] depending on actin filaments but also involving microtubules [51] to drive cellulose alignment [52].

In this study, we were able to follow and diversify the trafficking of the soluble vacuolar markers RFP-Chi and Aleu-GFP through the selective inhibition of the two cytoskeleton components actin and microtubules, mediated respectively by Cyt D and Tax. Cyt D is a cell-permeable fungal toxin that binds to the barbed end of actin filaments, inhibiting both the association and dissociation of subunits. This alkaloid, binding the actin F monomer, causes the disruption of actin filaments and inhibition of actin polymerization. Tax has been traditionally used to promote and stabilize tubulin polymerization in plants [53], causing the disorganization of the microtubule framework and detachments from membranes [54].

Through our experimental design, we confirmed here that RFP-Chi transits through an intermediate compartment different from the Golgi [16] or at least different from the compartments labeled by ERD2-YFP and CslA2-GFP. On the contrary, Aleu-GFP transits through the Golgi thanks to the well-known sorting pathway based on VRS1 recognition. The use of transient expression allowed a reasonable time-dependent resolution of the markers’ sorting. Results indicated that actin filaments and microtubules differently contributed to Golgi-dependent and Golgi-independent export from the ER.

Cyt D was more effective in preventing the export from ER of the Golgi marker CslA2-GFP (Figure 2D) than the export of RFP-Chi (Figure 1E). The effect on RFP-Chi distribution could be visualized in two different patterns due to long-term or short-term effects. After a long expression time, in the presence of inhibitors, the pattern was altered by both drugs, but inducing the formation of aberrant large compartments. Since large pro-vacuolar compartments are also generated in the normal pattern (Figure S3), the description of differences is difficult. The small intermediate compartments were hardly distinguishable from larger compartments that form with time. Several markers co-localized within these altered compartments. The complexity of the long-term pattern may be explained by the fact that TGN correct organization and function was altered by both inhibitors. Cyt D affected the Golgi contribution to the TGN maturation; Tax affected the ER contribution to TGN final identity. This was highlighted by the differentiated effect of inhibitors on ST52-mCherry. Cyt D appeared to block the marker at the Golgi/TGN level (Figure 2E), while Tax redistributed the marker in the ER (Figure 2H) through a still unknown mechanism.

Since the long-term distribution pattern of vacuolar markers is complex and overlaps in tobacco photosynthetic parenchyma cells with or without inhibitors, we analyzed in detail the short-term effect, especially on markers’ export from the ER, focusing on small compartments directly emerging from the ER in the early stage of sorting (Figure 4).

In the case of Aleu-GFP, the export from the ER leads to Golgi bodies but in the case of RFP-Chi it leads to “ERMEC”, the still uncharacterized intermediate compartments emerging from the ER as microtubule-dependent export compartments [16]. Our definition of intermediate compartments derives from the observation that they are more abundant during the early phases of transient expression, when the central vacuole is not yet intensely labeled. Some of the observed compartments were smaller than 2 micrometers; others grew larger but we assumed that, over 4–5 micrometers in diameter, these could be considered pro-vacuoles and no longer considered intermediate compartments. Figure 4 summarizes the behavior of small compartments labeled by the two vacuolar markers. Aleu-GFP labeled a large number of small compartments (presumably dictyosomes), while RFP-Chi-labeled compartments were much less abundant and mostly co-localized with Aleu-GFPs. These compartments were characterized by a very high mobility, making imaging challenging. During the first few hours after treatment with Cyt D, the small compartments were still co-localizing, but after 18–21 h, the number of RFP-Chi-labeled compartments not showing any GFP labeling was very much increased. We then hypothesized that actin was more important for the transport of Aleu-GFP, while RFP-Chi continued to accumulate in the intermediate compartments despite the defect in Golgi transport.

The treatment with Tax affected compartments’ motility too and also induced a significant and immediate increase in RFP-Chi-labeled compartments not showing any overlapping with GFP labeling at the Golgi. The number of these compartments did not increase with time, and we hypothesized that microtubules were required to make these compartments arise from the ER and merge with Golgi traffic, justifying the name “ERMEC”.

**Figure 8:**
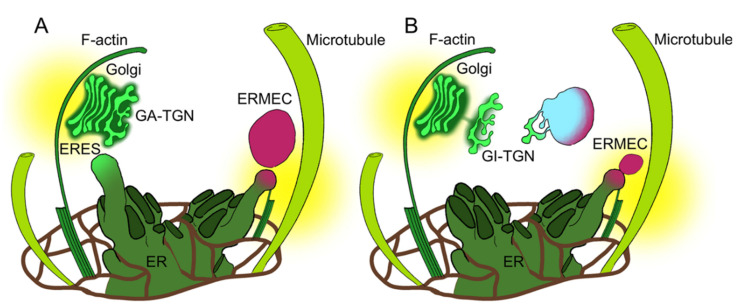
Schematization of endoplasmic reticulum (ER) export mechanisms associated with cytoskeleton elements: actin filaments (F-actin) and microtubules. (**A**) The Golgi arises from the ER export sites (ERES), moved by the association with actin filaments, and matures its *trans*-cisterna in a Golgi-associated *trans*-Golgi network (GA-TGN); at the same time, tubulin drives the formation of compartments (ERMEC) independently from the Golgi. (**B**) The Golgi-independent TGN (GI-TGN) fuses with ERMEC to allow vacuolar sorting. Both elements play a role in the process.

Thus, Golgi traffic of Aleu-GFP depends more on actin while ERMEC traffic of RFP-Chi depends more on tubulin. Figure 8 summarizes the interpretation of our results. The scheme suggests that the Golgi-independent TGN (GI-TGN) merges with the ERMEC to form a new specialized post-Golgi compartment or simply progresses in its maturation.

The in silico protein interaction network revealed that both Vti11 and Vti12, considered characteristic of the alternative sorting pathways [17], interact with SYP51, a SNARE that was recently found to be involved in specific traffic events to the vacuole, and which in turn was found to interact with NIP1.1 [18], found to be involved in direct export from the ER [7,18]. Interestingly these two proteins were proposed to regulate the cross-talk between Golgi-related and Golgi-independent traffic [18].

*Trans*-Golgi/TGN markers, ST52-mCherry and GFP:SYP51, were seen to be affected by both cytoskeleton targets. This once more confirms that the correct functioning of the cytoskeleton as a whole is required for the correct organization of the TGN.

The marker CslA2-GFP helped to evidence that the effect on the Golgi is not directly related to the effect of cytoskeleton defects on the TGN. As previously suggested by the study of the sorting of the RFP-Chi putative receptor, RMR1, a direct traffic from the ER, may have an influence on TGN functions [11]. Merging of different traffic pathways in the TGN may sound surprising if we do not consider the possibility that maturation of Golgi-independent TGNs may offer the opportunity to specialize these *post*-Golgi organelles into very specialized entities. We cannot exclude that ERMEC itself is a specialized form of TGN. Despite their potential specialization, GI-TGNs would always need the contribution of different trafficking machineries, including direct traffic from the ER. Until the TGN maturation process reveals its potential diversification, it will be difficult to fully elucidate all endomembrane traffic pathways.

## Supplementary Materials

The following are available online at https://www.mdpi.com/article/10.3390/membranes11040299/s1, Figure S1: (A) CslA-GFP, (B) RFP-Chi and (C) merge distributions in the presence of Cyt D after longer treatment (15 h). Scale bar = 10 µm. Figure S2: (A) CslA-GFP, (B) RFP-Chi and (C) merge distributions in the presence of Taxol (Tax) after longer treatment (15 h). Scale bar = 10 µm. Figure S3: RFP-Chi and Aleu-GFP after 24 hours (A–C) co-localizing in small compartments; (D–F) distributing in vacuoles with RFP-Chi persistent in the endoplasmic reticulum (ER) and pro-vacuoles. (G–I) Treated with Cyt D for 24 hours, with small compartments no longer co-localizing; (J–L) both markers arriving to the vacuoles less efficiently, co-localizing in probably aberrant pro-vacuoles; (M–O) Tax treatment has a lesser effect on Aleu-GFP than on RFP-Chi. Scale bar = 10 µm.
