# Supplementary material for: Actin and Microtubules Differently Contribute to Vacuolar Targeting Specificity during the Export from the ER
Source: Membranes (Basel). 2021 Apr 20;11(4):299. doi: 10.3390/membranes11040299 (PMC8074374; doi:10.3390/membranes11040299)
Supplement: Supplementary file 1 [file membranes-11-00299-s001.zip › membranes-1180915-supplementary.pdf]

membranes

Supplementary Materials: Actin and Microtubules Differently Contribute to Vacuolar Targeting Specificity During the Export from the ER

Monica De Caroli [1], Fabrizio Barozzi [1,2], Luciana Renna [3], Gabriella Piro [1] and Gian-Pietro Di Sansebastiano [1,*]

[1] DISTEBA (Department of Biological and Environmental Sciences and Technologies), University of Salento, Campus ECOTEKNE, 73100 Lecce, Italy; monica.decaroli@unisalento.it (M.D.C.); barozzi.fabrizio@virgilio.it (F.B.); gabriella.piro@unisalento.it (G.P.); gp.disansebastiano@unisalento.it (G.-P.D.S.)
[2] Department of Plant Physiology, Faculty of Biology, Chemistry and Earth Sciences, University of Bayreuth, Universitätsstraße 30, D-95447 Bayreuth
[3] Department of biology, University of Florence, 50121 Firenze, Italy; luciana.renna@unifi.it
* Correspondence: gp.disansebastiano@unisalento.it; Tel.: +39-0832-298-714

SUMMARY

Number of pages: 3
Number of figures: 3

Citation: De Caroli, M.; Barozzi, F.; Renna, L.; Piro, G.; Sansebastiano, G.-P. Actin and microtubules differently contribute to vacuolar targeting specificity during the export from the ER. *Membranes* **2021**, *11*, 299. https://doi.org/10.3390/membranes11040299

Academic Editor: Cinzia Progida

Received: 28 March 2021
Accepted: 14 April 2021
Published: 23 April 2021

Publisher's Note: MDPI stays neutral with regard to jurisdictional claims in published maps and institutional affiliations.

Copyright: © 2021 by the authors. Licensee MDPI, Basesl, Switzerland. This article is an open access article distributed under the terms and conditions of the Creative Commons Attribution (CC BY) license (http://creativecommons.org/licenses/by/4.0/).

S

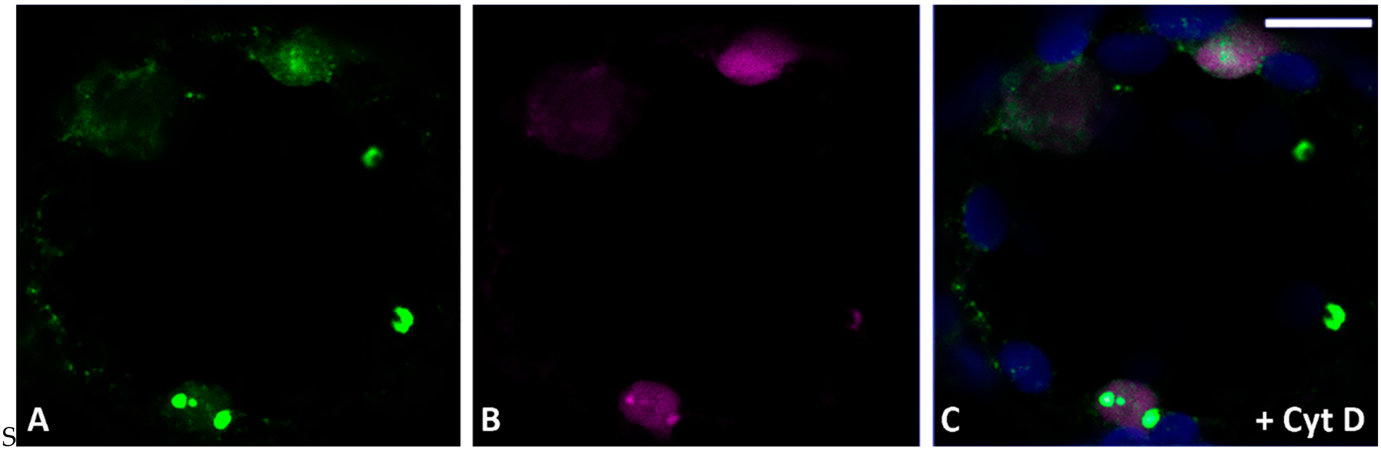

Figure S1. (**A**) CslA-GFP, (**B**) tobacco Chitinase A (RFP-Chi) and (**C**) merge distributions in the presence of Cytochalasin D (Cyt D) after longer treatment (15 h). Scale bar = 10 µm.

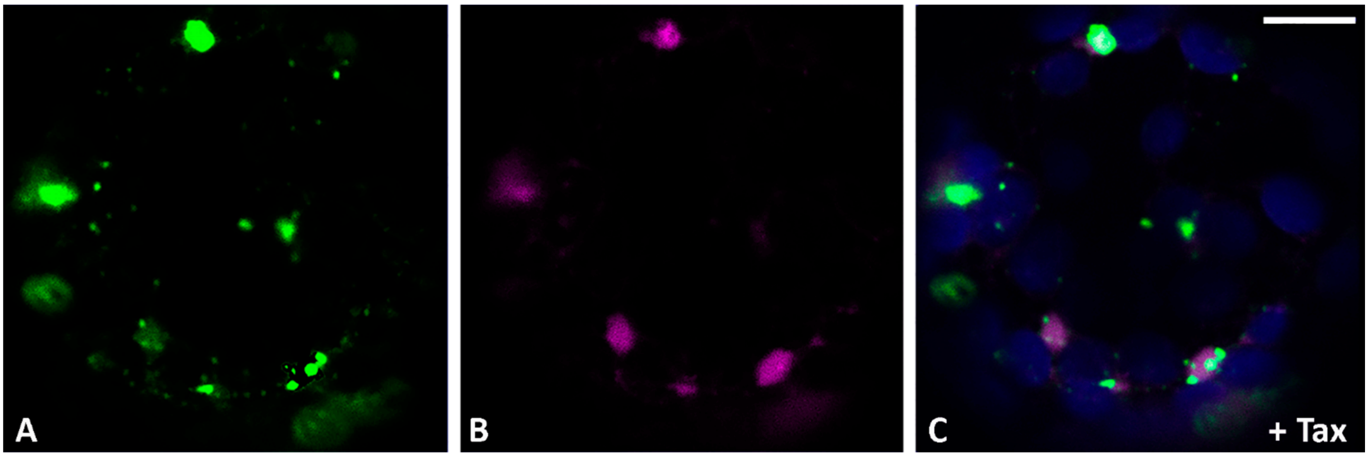

Figure S2. (**A**) CslA-GFP, (**B**) RFP-Chi and (**C**) merge distributions in the presence of Taxol (Tax) after longer treatment (15 h). Scale bar = 10 µm.

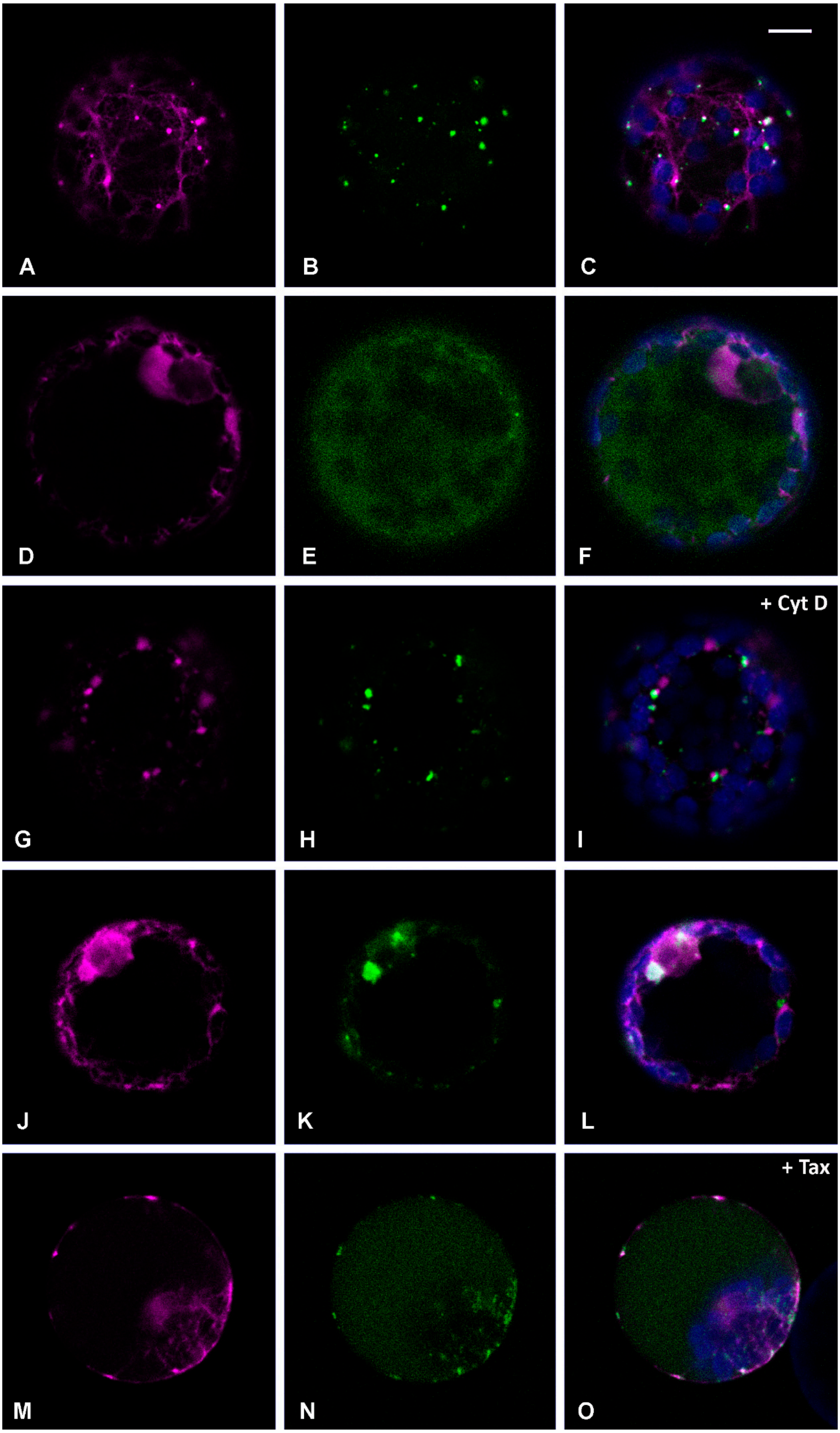
A
B
C
D
E
F
+ Cyt D
G
H
I
J
K
L
+ Tax
M
N
O

Figure S3. RFP-Chi and Aleu-GFP after 24 h (**A–C**) co-localizing in small compartments; (**D–F**) distributing in vacuoles with RFP-Chi persistent in the endoplasmic reticulum (ER) and pro-vacuoles. (**G–I**) Treated with Cyt D for 24 h, with small compartments no longer co-localizing; (**J–L**) both markers arrive to the vacuoles less efficiently, co-localizing in probably aberrant pro-vacuoles. (**M–O**) Tax treatment has a lesser effect on Aleu-GFP than on RFP-Chi. Scale bar = 10 µm.

About Supplemental Figure 3: The evaluation of interfering effects of cytochalasin D (Cyt D) and Taxol (Tax) on vacuolar transport is difficult when the effect is not drastically different. In the case of cytoskeleton alteration, both markers finally suffer an effect. Moreover, the fluorescence in the central vacuole may be influenced by other parameters such as pH, which may possibly be altered as well. In the figure, we see two examples of what we can see in control conditions. A) Small compartments nearly completely co-localizing and B) Aleu-GFP mostly in the central vacuole and tobacco chitinase A (RFP-Chi) reaching the central vacuole more slowly, persistent in the ER and accumulated in small pro-vacuoles as previously shown for the GFP variant [55, 15]. Cyt D induces the complete separation of small compartments and disturbs the arrival in the vacuole of both markers, causing the co-localization of both in medium size pro-vacuoles, which, in this case, may probably be aberrant compartments. Tax seems to be more specific in slowing down RFP-Chi sorting, since Aleu-GFP seems to normally arrive to the central vacuole. Despite these observations, it was anyhow difficult to quantify these parameters because cells appeared all somehow affected by the inhibitors. Thus, we preferred to concentrate the observation on the early steps of sorting.

References

1. Di Sansebastiano, G.; Paris, N.; Marc-Martin, S.; Neuhaus, J. Specific accumulation of GFP in a non-acidic vacuolar compartment via a C-terminal propeptide-mediated sorting pathway. *Plant J.* **1998**, *15*, 449–457, doi:10.1046/j.1365-313x.1998.00210.x.
2. Di Sansebastiano, G.P.; Paris, N.; Marc-Martin, S.; Neuhaus, J.-M. Regeneration of a Lytic Central Vacuole and of Neutral Peripheral Vacuoles Can Be Visualized by Green Fluorescent Proteins Targeted to Either Type of Vacuoles. *Plant Physiol.* **2001**, *126*, 78–86, doi:10.1104/pp.126.1.78.
